# Pulmonary hypertension in familial Mediterranean fever: consequence or coincidence?

**DOI:** 10.1186/1546-0096-13-S1-O41

**Published:** 2015-09-28

**Authors:** A Sargsyan, M Narimanyan

**Affiliations:** 1 Yerevan State Medical University, Internal Medicine, Yerevan, Armenia; 2 Yerevan State Medical University, Family Medicine, Yerevan, Armenia

## Objectives

FMF is the most common autoinflammatory disease characterized by recurrent febrile polyserositis. The gravest consequence of FMF is nephropatic amyloidosis of AA type, which may progress to affect other organs, including the lungs[^1^]. Pulmonary hypertension (PH) in FMF related amyloidosis is rare; only a few cases have been reported so far[^2^,^3^]. We aim to elucidate development of PH in FMF in Armenian patients group.

## Methods

80 FMF patients without amyloidosis (mean age 33.6±11.8, male/female 45/35) and 75 FMF-amyloidosis patients (37.8±7.4, 42/33) were included in the study and followed prospectively for 3 years. All patients had recurrent pleuritis except of three phenotype II patients. Selected patients were homo/heterozygous for the M694V(n=122), M680I(28), V726A(4) and E148Q(1) mutations. Chest X-ray, pulmonary function test, ECG, transthoracic Doppler echocardiography (TTE) and CT-scan were carried out. Hb, ESR, leucocytes, fibrinogen, CRP, SAA, creatinine and capillary blood gases were measured. All patients were attack-free under colchicine treatment at the time of the study except of two hemodialysis patients. We considered patients to have PH if their estimated pulmonary artery systolic pressure (PASP) was >35 mm Hg as measured by TTE.

## Results

(6%) FMF patients without amyloidosis and 9 (12%) with amyloidosis were diagnosed having PH (male/female 5/9). The median age at the diagnosis of PH was 48 years (range, 36-72). The median FMF duration at the time of PH diagnosis was 36 (1-60) years. All patients had symptoms related to PH: exertional dyspnea and fatigue (14 patients), chest pain (10), hepatomegaly (7), anorexia and weight loss (7), peripheral edema (6), ascites (5), cough (3), palpitation (3) and syncope (1). 4 patients had palpable right ventricular lift and 6 had increased intensity of P2 or splitting of S2. TTE data were as following: the median ejection fraction was 50% (20-60), the median PASP was 40 mm Hg (36-64). 9 patients had right ventricular dilatation and/or hypertrophy, tricuspid regurgitation of different degree, and 3 patients had pericardial effusion (90, 120, 150ml). Chest X-ray findings were abnormal in 11 patients (9 FMF-amyloidosis and 2 without amyloidosis) and showed opacities, hilar adenopathy, pleural thickening and pleural effusion. The other 3 patients had pleural adhesions. Chest CT scans findings were suggestive for cardiac and pulmonary amyloidosis (interstitial reticulonodular infiltrates) in 8 patients. PFT revealed restrictive pattern in 10 patients, restriction with mild obstruction in 2 patients and lung volumes were normal in 1 patient without amyloidosis.

Patients were not treated for their PH. Three patients died of cardiac complications (congestive heart failure), they had impaired kidney function as well, and one amyloidosis patient died because of uremia. On autopsy amyloid deposits in kidney, spleen, liver, heart and lungs were found in three cases. The median time to death after the diagnosis of PH was 470 days (range 135-1095).

Lab tests were as following in FMF-amyloidosis patients group vs group without it (mean±SD): CRP 17.74±13.74 mg/L vs 11,88±13.79 mg/L and SAA 33±66.6 mg/L vs 5.25±4,45mg/L (p<0,0001). PO_2_83.6±8.95 mmHg, PCO_2_39.4±3.6 mmHg, O_2_Sat 94.6±3.38% vs. PO_2_74±11.36, PCO_2_35.3±4.5, O_2_Sat 90.1±10.26% (P<0.0001).

## Conclusions

Patients with FMF may develop PH late in the disease process with resultant right-sided heart failure. The prognosis of AA amyloidosis depends on the degree of renal dysfunction at presentation and whether the underlying inflammatory disease can be effectively suppressed[^4^]. We speculate that PH in FMF is a consequence of ongoing inflammation and amyloidosis with subsequent early vascular alteration. This may explain PH development in the absence of severe intravascular amyloid deposits. PH develops in most untreated patients and in those who are on hemodialysis. In published case reports[^2^,^3^] the prognosis of FMF patients with PH and amyloidosis was poor. Our data are in accordance with them; PH in FMF may have a fatal course. We conclude that PH should be considered in FMF patients with dyspnea, fatigue or fluid overload, especially in individuals with amyloidosis. If present, PH should be monitored and treated.
